# A clinical scoring system for the early identification of superinfections in patients with severe fever with thrombocytopenia syndrome: An ambispective multicentre cohort study

**DOI:** 10.1371/journal.pntd.0014696

**Published:** 2026-09-10

**Authors:** Yu-Yao Li, Jian-Kang Zhang, Chun Zhang, Zhi-Ping Pan, Xiao Liu, Meng-Yu Liu, Qiang Chen, Yuan-Yuan Zhang, Yuan Jiang, Xu-Ya Yuan, Si-Yang Liu, Xiang Li, Fei-Dan Yu, Yu-Feng Gao, Jun Cheng, Qin-Xiu Xie, Jian-Guo Rao, Li-Yu Zhu, Zhen-Jun Liu, Ying Ye, Jia-Bin Li, Li-Fen Hu

**Affiliations:** 1 Department of Infectious Diseases, the First Affiliated Hospital of Anhui Medical University, Hefei, Anhui, China; 2 Department of Infectious Diseases, Lu’an People’s Hospital, Lu’an, Anhui, China; 3 Department of Infectious Diseases, Chaohu Hospital of Anhui Medical University, Chaohu, Anhui, China; 4 Department of Infectious Diseases, Anqing Municipal Hospital, Anqing, Anhui, China; 5 Department of Cardiology, Sun Yat-sen Memorial Hospital of Sun Yat-sen University, Guangzhou, Guangdong, China; 6 Anhui Province Key Laboratory of Infectious Diseases, Anhui Medical University, Hefei, China; 7 Department of Infectious Diseases, Public Health Clinical Center of Anhui Province, Hefei, China; 8 Programme in Emerging Infectious Diseases, Duke-National University of Singapore Medical School, Singapore, Singapore; NIAID Integrated Research Facility, UNITED STATES OF AMERICA

## Abstract

**Background:**

Superinfection is a major contributor to mortality in patients with severe fever with thrombocytopenia syndrome (SFTS). However, early identification remains challenging because detection of the pathogen causing the superinfection is often delayed.

**Objective:**

This study aims to develop a clinical scoring system for the early identification of superinfection in SFTS patients.

**Methods:**

Among 1942 SFTS patients from 4 hospitals in China, a retrospective cohort (2014–2024) was used for model development, with 1182 patients from 2 hospitals split into training (n = 823) and internal validation (n = 359) sets, and 475 patients from 2 additional hospitals for external validation sets. Four machine learning algorithms were evaluated, with the optimal model converted into a simplified scoring scale. Finally, a prospective cohort (n = 285, 2025) evaluated the real-world performance. Model efficacy was comprehensively assessed using the area under the receiver operating characteristic curve (AUROC), calibration curves, decision curve analysis (DCA), sensitivity, and specificity.

**Results:**

Among the 36 variables, 8 predictors (age, ALB, AST, BUN, GLU, CRP, HGB, and expectoration) for superinfection were identified by ensembling five machine learning algorithms. The RF, XGBoost, LightGBM, and LR prediction models were constructed using the 8 predictors. LR demonstrated optimal performance, with an AUROC of 0.839 (95% CI: 0.797–0.880) in the internal validation set and 0.805 (95% CI: 0.764–0.847) in the external validation set. In the real-world prospective study, the model maintained high predictive efficacy (AUROC: 0.854). Finally, the model’s nomogram was simplified into a novel 3-tiered risk scoring scale to enhance clinical applicability.

**Conclusions:**

Our research developed a dynamic, early diagnostic tool that could improve real-time prediction of superinfection risk at the bedside and enhance antimicrobial stewardship.

## Author summary

Severe fever with thrombocytopenia syndrome (SFTS) is a life-threatening tick-borne disease. It is characterized by a high incidence of superinfections, which substantially increase the risk of mortality. Unfortunately, delayed identification of superinfections often leads to worse outcomes or inappropriate antibiotic use in SFTS patients. In this multicenter study spanning 12 years, we developed a data-driven clinical prediction model designed to stratify the risk of superinfection in SFTS patients using four machine learning algorithms. The model’s performance and clinical utility were demonstrated through receiver operating characteristic curve analysis, calibration assessment, decision curve analysis, sensitivity, and specificity. Logistic regression was identified as the optimal algorithm, with strong predictive accuracy across internal and external validation. Its efficacy was further confirmed in a prospective real-world cohort, which achieved an excellent discriminatory power. Ultimately, the model was simplified into a three-tier risk scoring system, enabling rapid bedside assessment of superinfection risks without the need for complex computational tools. Overall, by identifying key clinical indicators, our model provides clinicians with an effective tool for the rapid identification of superinfection risk in SFTS patients using easily accessible predictors.

## Introduction

Severe fever with thrombocytopenia syndrome (SFTS) caused by a novel Bunyavirus is a newly emerging infectious disease. The novel Bunyavirus, SFTS virus (SFTSV), is transmitted mainly through tick bites but can also be transmitted between humans [1,2]. SFTS is a severe disease with an acute onset and has been increasingly reported in China and other Asian countries [3–5]. The case fatality rate (CFR) of hospitalized patients with SFTS has been reported to be 10% –30% [2,6–8].

Superinfection contributes significantly to the high CFR of SFTS [9], with reported incidence among patients ranging from 27.4% to 42.9% [10,11]. While delayed treatment is associated with poor prognosis and increased mortality, clinicians often face uncertainty regarding the diagnosis of superinfection. Clinicians are facing a great challenge in determining which SFTS patients should receive antibiotic treatment. Therefore, early diagnosis of superinfection is crucial; however, pathogen identification is often delayed, hindering antimicrobial stewardship in SFTS patients.

Based on the inherent challenges of traditional pathogen detection including sample contamination, false negatives, and reporting delays, artificial intelligence is emerging as a critical breakthrough for achieving early and precise diagnosis [12]. Machine Learning(ML)-based tools are capable of deeply integrating and analyzing multi-source, real-time data from patient clinical symptoms, laboratory indicators, and medical images, thereby helping clinicians achieve early warning and timely diagnosis [13].

To embody this ML-driven approach in SFTS with superinfection management, we collected retrospective data from all consecutive SFTS patients who were treated at 4 hospitals in 4 cities over a period of 11 years. These data were utilized for model construction to predict superinfection of SFTS patients using machine learning algorithms. Furthermore, the model’s clinical utility was validated in a prospective, real-world cohort to evaluate its capacity for the early identification of superinfections. The validated, model-based diagnostic tool enables clinicians to conduct real-time superinfection risk assessments of SFTS patients at the bedside.

## Methods

### Ethical statement

This study followed the ethical guidelines of the Helsinki Declaration and was approved by the Ethics Committee of Anhui Medical University (Approval No. 20200980). Written informed consent was obtained from all participants in the prospective cohort of this study, while the requirement for informed consent was waived for the retrospective cohort.

### Study design and population

This study employed an ambispective, multicenter cohort design conducted in two sequential phases to develop and validate a prediction model for superinfection in SFTS patients. During the retrospective phase (January 2014 to December 2024), data on laboratory-confirmed SFTS patients were collected over a consecutive eleven-year period from four hospitals in Anhui Province. These centres comprised the First Affiliated Hospital of Anhui Medical University in Hefei, Chaohu Affiliated Hospital of Anhui Medical University in Chaohu, Lu’an People’s Hospital in Lu’an, and Anqing Municipal Hospital in Anqing. Together, they provided a comprehensive dataset for the training, internal validation, and external validation sets. In the subsequent prospective phase (March to September 2025), SFTS patients were enrolled at the Hefei and Lu’an hospitals to constitute a prospective real-world validation set.

Patients were eligible for inclusion if they had a laboratory-confirmed SFTSV infection and complete clinical records for model development. To minimize confounding variables, we excluded patients with pre-existing chronic infections (such as tuberculosis or chronic intestinal infections), haematological diseases, or malignancies. Patients with insufficient documentation to confirm the presence or absence of superinfections were excluded.

### Definition and data collection

Laboratory-confirmed SFTS was defined on the basis of the criteria used for the diagnosis and treatment of SFTS [14]. Respiratory tract infection was determined by the presentation of clinical symptoms, imaging abnormalities, and laboratory indicators, including the presence of pathogens [15]. Bloodstream infection and urinary tract infection were diagnosed by assessing clinical symptoms and pathogens in blood or urine samples [16]. Cholecystitis was defined based on clinical symptoms and imaging abnormalities [17]. Pathogens were identified through blood culture, sputum culture, next-generation sequencing (NGS), or polymerase chain reaction (PCR). All identified pathogens were consistent with the post-SFTS onset period and clinically relevant to the site of infection. In this context, the term “superinfection” refers specifically to these secondary bacterial or fungal complications arising during the course of SFTS.

The primary outcome of the study was superinfection occurring within 30 days of disease onset. We collected data for the first three days of hospitalization, including clinical manifestations, laboratory data, treatment regimens, underlying diseases, and outcomes, from electronic health records, and all data were cross-checked by trained researchers. The proportion of missing values for all variables was less than 5%. No missing data were observed in categorical variables. For continuous variables with a small number of missing values, mean imputation was performed. Superinfection was assessed only when it occurred on or after hospital day 4, thereby improving the model’s utility for early clinical decision-making.

### Predictors selection and model development

**Fig 1:**
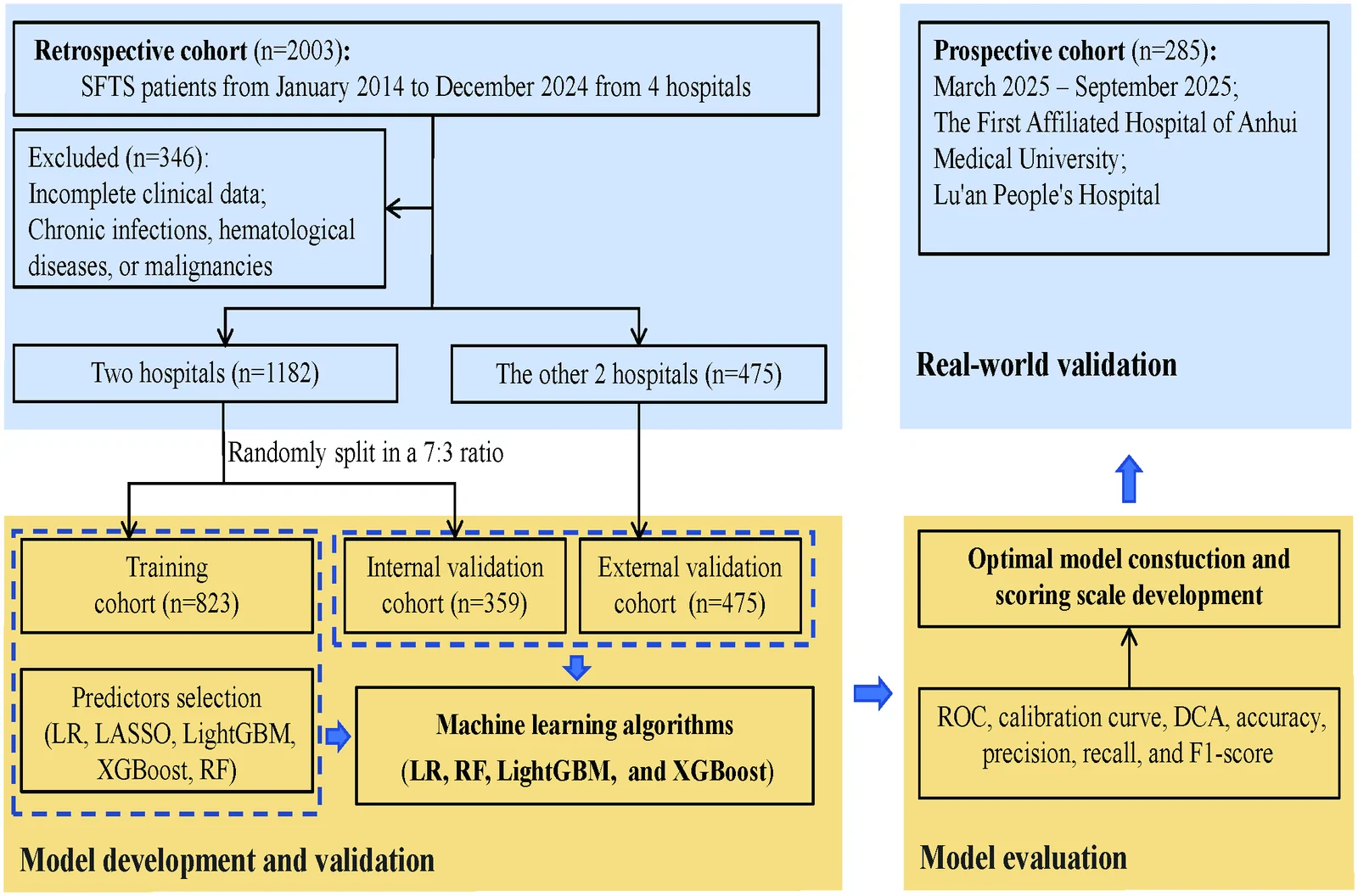
Flow charts of patient recruitment and exclusion criteria. To construct a diagnostic model for predicting superinfection in SFTS patients. Abbreviations: SFTS, severe fever with thrombocytopenia syndrome; LASSO, least absolute shrinkage and selection operator regression; LR, Logistic Regression; LightGBM, Light Gradient Boosting Machine; XGBoost, Extreme Gradient Boosting.

As shown in Fig 1, the 1182 SFTS patients from the First Affiliated Hospital of Anhui Medical University and Anqing Municipal Hospital between 2014 and 2024 were randomly divided into a training set (n = 823) and an internal validation set (n = 359) at a ratio of 7:3. Meanwhile, stratified random sampling was conducted based on whether superinfection occurred. A total of 36 variables covering demographics, underlying diseases, clinical features, laboratory findings, and glucocorticosteroid administration, were included in the training dataset to identify independent predictors of superinfection.

In the training set (n = 823), univariate logistic regression was first applied to all 36 candidate variables; 28 variables with *p* < 0.05 were retained for the Least Absolute Shrinkage and Selection Operator (LASSO) regression [18]. The 36 variables were then processed in parallel using three tree-based ML algorithms: Random Forest (RF) [19], Light Gradient Boosting Machine (LightGBM) [20], and Extreme Gradient Boosting (XGBoost) [21]. The complete hyperparameter search spaces and the values used for model fitting are provided in S4 Table. LASSO retained 14 variables, and the top 15 variables ranked by feature importance were extracted from each of the three tree-based models. The final feature set was then defined as the intersection of two groups: the variables retained by LASSO, and the union of the top-15 variables from RF, XGBoost, and LightGBM. This conservative cross-algorithmic strategy yielded 8 robust predictors, reduced algorithm-specific bias, and minimised the risk of overfitting [22].

### Model validation and evaluation

Model validation was performed in three sequential stages. First, internal validation was performed on a 30% random subset of the 1,182 retrospective patients from Hefei and Anqing, allocated by stratified random sampling on the outcome variable to preserve event proportions. Second, external validation was performed on an independent cohort of 475 patients from Chaohu and Lu’an, two hospitals not represented in the training data, in order to assess geographic and institutional generalizability. Third, prospective real-world validation was performed in 285 consecutive SFTS patients enrolled between March and September 2025 at the Hefei and Lu’an hospitals, using the same data-collection protocol as the retrospective cohorts.

Model discriminative ability was evaluated using the AUROC with 95% confidence intervals. Calibration between predicted risks and observed outcomes was assessed via calibration curves, and overall predictive accuracy was quantified using the Brier score. The DCA was performed to determine the net clinical benefit and practical utility of the model across different risk thresholds. Diagnostic performance was further characterized by sensitivity, specificity, positive predictive value (PPV), and negative predictive value (NPV). All models were comprehensively evaluated via internal and external validation, and the optimal model was additionally validated in a real-world cohort.

### Comparison of nomogram and simplified scoring scale

For the convenience of clinical application, the final predictive indicators were divided into hierarchical variables and rescored based on the nomogram. A new scoring scale ranging from 0 to 13 points was ultimately developed. The simplified scoring scale uses a binary cut-off of 6.5, corresponding to a total score of ≥ 7. This cut-off was determined by maximising Youden’s index in the training cohort and was applied unchanged to all subsequent validation cohorts. The three-tier classification combined the empirical tertile structure of the score distribution with anchoring at the Youden-optimal cut-off. Both threshold systems were derived exclusively in the training cohort and were not re-tuned in any validation cohort.

### Statistical analysis

Normally distributed quantitative variables were presented as the mean (standard deviation, SD) and compared using the Student’s t-test, while non-normally distributed variables were presented as the median (interquartile range, IQR) and analyzed using the Mann–Whitney U test. Categorical variables were expressed as frequencies and proportions and compared using the Pearson chi-square test or Fisher’s exact test, as appropriate. Statistical analysis was performed using R software (version 4.4.3), and a *p-*value < 0.05 was considered statistically significant.

## Results

### Characteristics of the study population

Among the 2288 SFTS patients from 2014 to 2025, 1942 were included in the study (Fig 1). Of these, 652 (33.57%) had superinfection, including 25.18% with respiratory tract infection, 5.77% with bloodstream infection, 1.54% with urinary tract infection, and 0.42% with cholecystitis. The CFR in the superinfection group has been increased by 2.3 times compared to the non-superinfection group (31.13% *vs.* 13.57%, *p* < 0.001) (Table 1).

**Table 1:** Comparisons of clinical and laboratory characteristics between SFTS patients with superinfection and non-superinfection.

Characteristics	Overall (N = 1,942)	Non-superinfection (N = 1,290)	Superinfection(N = 652)	*p*-value
**Clinical characteristics, treatment, symptoms, or signs**				
Age, year	67.00 (57.00, 74.00)	65.00 (55.00, 72.00)	71.00 (64.00, 76.00)	<0.001
Male, N (%)	826 (42.53%)	543 (42.09%)	283 (43.40%)	0.615
Systolic pressure,(mmHg)	114.00 (101.00, 126.00)	114.00 (102.00, 125.00)	113.00 (100.00, 126.00)	0.403
Glucocorticosteroids, N (%)	648 (33.37%)	363 (28.14%)	285 (43.71%)	<0.001
Fever, N(%)	1,887 (97.17%)	1,245 (96.51%)	642 (98.47%)	0.021
Gastrointestinal symptoms, N (%)	1,220 (62.82%)	783 (60.70%)	437 (67.02%)	0.007
Lymphadenopathy, N (%)	509 (26.21%)	317 (24.57%)	192 (29.45%)	0.024
Expectoration, N (%)	376 (19.36%)	132 (10.23%)	244 (37.42%)	<0.001
Bleeding, N (%)	313 (16.12%)	138 (10.70%)	175 (26.84%)	<0.001
Cough, N (%)	493 (25.39%)	209 (16.20%)	284 (43.56%)	<0.001
Limb tremor, N (%)	289 (14.88%)	144 (11.16%)	145 (22.24%)	<0.001
Confusion, N (%)	419 (21.58%)	177 (13.72%)	242 (37.12%)	<0.001
Coma, N (%)	159 (8.19%)	60 (4.65%)	99 (15.18%)	<0.001
**History**				
DM, N (%)	194 (9.99%)	100 (7.75%)	94 (14.42%)	<0.001
COPD, N (%)	62 (3.19%)	31 (2.40%)	31 (4.75%)	0.008
**Laboratory parameters**				
GLU, (mmol/L)	7.14 (5.79, 9.32)	6.66 (5.50, 8.78)	8.28 (6.38, 11.49)	<0.001
ALB, (g/L)	30.90 (27.30, 34.80)	32.60 (29.00, 36.00)	27.90 (25.05, 31.40)	<0.001
GLO, (g/L)	27.20 (23.70, 31.40)	26.60 (23.30, 30.80)	28.30 (24.70, 33.00)	<0.001
TBIL, (umol/L)	12.69 (8.80, 21.52)	11.74 (8.40, 18.89)	15.15 (9.76, 27.53)	<0.001
ALT, (U/L)	79.00 (45.00, 138.00)	75.00 (42.00, 129.00)	91.00 (54.00, 155.00)	<0.001
AST, (U/L)	165.00 (84.00, 326.00)	139.00 (72.00, 256.00)	232.00 (117.00, 470.00)	<0.001
LDH, (U/L)	682.00 (399.00, 1,295.00)	574.00 (360.00, 1,021.00)	986.00 (543.50, 1,906.00)	<0.001
BUN, (mmol/L)	6.40 (4.72, 8.89)	5.89 (4.43, 7.93)	7.73 (5.56, 11.64)	<0.001
Cr, (umol/L)	73.00 (59.30, 95.00)	69.00 (57.20, 88.10)	81.40 (64.80, 115.43)	<0.001
CK, (U/L)	445.50 (183.00, 1,101.00)	373.50 (153.00, 946.00)	595.00 (278.50, 1,336.50)	<0.001
CKMB, (U/L)	21.00 (12.00, 36.40)	19.00 (11.00, 32.20)	25.00 (14.00, 44.87)	<0.001
K, (mmol/L)	3.48 (3.15, 3.84)	3.50 (3.18, 3.82)	3.44 (3.03, 3.87)	0.004
Na, (mmol/L)	133.50 (130.60, 136.50)	133.90 (131.00, 136.90)	132.70 (129.70, 135.80)	<0.001
CRP, (mg/L)	5.69 (1.15, 19.52)	3.38 (1.00, 11.00)	13.27 (4.07, 41.05)	<0.001
WBC, (×10^9^/L)	2.48 (1.57, 4.06)	2.44 (1.54, 3.86)	2.57 (1.62, 4.43)	0.033
Neutrophil, (×10^9^/L)	1.28 (0.78, 2.27)	1.20 (0.74, 2.11)	1.40 (0.86, 2.51)	<0.001
HGB, (g/L)	118.00 (103.00, 130.00)	121.00 (108.00, 132.00)	110.00 (92.00, 126.00)	<0.001
PLT, (×10^9^/L)	46.00 (31.00, 65.00)	51.00 (35.00, 70.00)	34.00 (24.50, 52.00)	<0.001
APTT, (Sec)	49.50 (41.30, 60.10)	48.20 (40.40, 57.60)	53.50 (44.65, 68.10)	<0.001
FIB, (g/L)	2.78 (2.32, 3.40)	2.74 (2.31, 3.26)	2.83 (2.33, 3.64)	0.005
DD, (ug/mL)	2.50 (1.37, 4.99)	2.33 (1.18, 4.20)	3.42 (1.86, 6.53)	<0.001
**Death, N (%)**	378 (19.46%)	175 (13.57%)	203 (31.13%)	<0.001

Abbreviations: DM, diabetes mellitus; COPD, chronic obstructive pulmonary disease; GLU, glucose; ALB, albumin; GLO, globulin; TBIL, total bilirubin; ALT, alanine aminotransferase; AST, aspartate aminotransferase; LDH, lactate dehydrogenase; BUN, urea nitrogen; Cr, creatinine; CK, creatinine kinase; CK-MB, creatine kinase MB; K, potassium; Na, sodium; CRP, C-reactive protein; WBC, white blood cell; HGB, hemoglobin; PLT, platelet count; APTT, activated partial thromboplastin time; FIB, fibrinogen; DD, d-dimer.

The superinfection group was significantly older and more likely to have glucocorticosteroid use (43.71%), diabetes mellitus (DM) (14.42%), chronic obstructive pulmonary disease (COPD) (4.75%), fever (98.47%), lymphadenopathy (29.45%), gastrointestinal symptoms (67.02%), bleeding (26.84%), cough (43.56%), expectoration (37.42%), limb tremor (22.24%), confusion (37.12%), or coma (15.18%) (*p* < 0.05) (Table 1). Compared to non-superinfection patients, patients with superinfection exhibited significantly lower levels of albumin (ALB: 27.90 g/L, [IQR 25.05, 31.40]), haemoglobin (HGB: 110.00g/L, [IQR 92.00, 126.00]), potassium (K: 3.44mmol/L, [IQR 3.03, 3.87]), sodium (Na: 132.70mmol/L, [IQR 129.70, 135.80]) and platelet count (PLT: 34.00 × 10^9^/L, [IQR 24.50, 52.00]) (*p* < 0.05). Conversely, the levels of glucose (GLU: 8.28mmol/L, [IQR 6.38, 11.49]), C-reactive protein (CRP: 13.27mg/L, [IQR 4.07, 41.05]), globulin (GLO: 28.30g/L, [IQR 24.70, 33.00]), total bilirubin (TBIL: 15.15umol/L, [IQR 9.76, 27.53]), alanine aminotransferase (ALT: 91.00U/L, [IQR 54.00, 155.00]), aspartate aminotransferase (AST: 232.00U/L, [IQR 117.00, 470.00]), urea nitrogen (BUN: 7.73mmol/L, [IQR 5.56, 11.64]), creatinine (Cr: 81.40umol/L, [IQR 64.80, 115.43]), lactate dehydrogenase (LDH: 986.00U/L, [IQR 543.50, 1,906.00]), creatinine kinase (CK: 595.00U/L, [IQR 278.50, 1,336.50]), creatine kinase MB (CK-MB: 25.00, [IQR 14.00, 44.87]), fibrinogen (FIB: 2.83g/L, [IQR 2.33, 3.64), and D-dimer (DD: 3.42ug/mL, [IQR 1.86, 6.53]); the neutrophil count (N: 1.40 × 10^9^/L, [IQR 0.86, 2.51]); and the activated partial thromboplastin time (APTT: 53.50s, [IQR 44.65, 68.10]) were significantly higher in the superinfection group (*p* < 0.05) (Table 1).

### Microbiological profile associated with superinfection in SFTS patients

Among the 652 SFTS patients diagnosed with superinfection, a total of 681 bacterial and fungal isolates were identified, with some patients presenting with more than one microorganism. As shown in S1 Fig, 223 (34.20%) isolates of Gram-negative bacilli, including 67 *Klebsiella pneumoniae*, 36 *Escherichia coli*, 41 *Acinetobacter*, 30 *Pseudomonas aeruginosa*, 28 *Stenotrophomonas maltophilia*, 12 *Enterobacter cloacae*, 5 *Haemophilus influenzae*, and 1 isolate each of *Salmonella Choleraesuis*, *Proteus mirabilis*, *Shigella*, and *Citrobacter freundii* were detected. We also detected 137 (21.01%) isolates of Gram-positive cocci, comprising 92 *Staphylococcus*, 25 *Streptococcus*, 15 *Enterococcus*, 3 *Bacillus cereus*, and 2 *Micrococcus luteus*. Additionally, 312 (44.78%) isolates of fungi were identified, including 203 *Aspergillus* (119 *A. fumigatus* and 59 *A. flavus*, 25 other *Aspergillus*), 106 *Candida*, 1 isolate each of *Mucor*, *Alternaria alternata,* and *Fusarium solani*. Besides, 9 patients were diagnosed with *Mycoplasma pneumoniae* infection. The novel finding was that 4 patients without a history of tuberculosis were diagnosed with active *Mycobacterium tuberculosis* infection.

### Early predictors selection for superinfection

**Fig 2:**
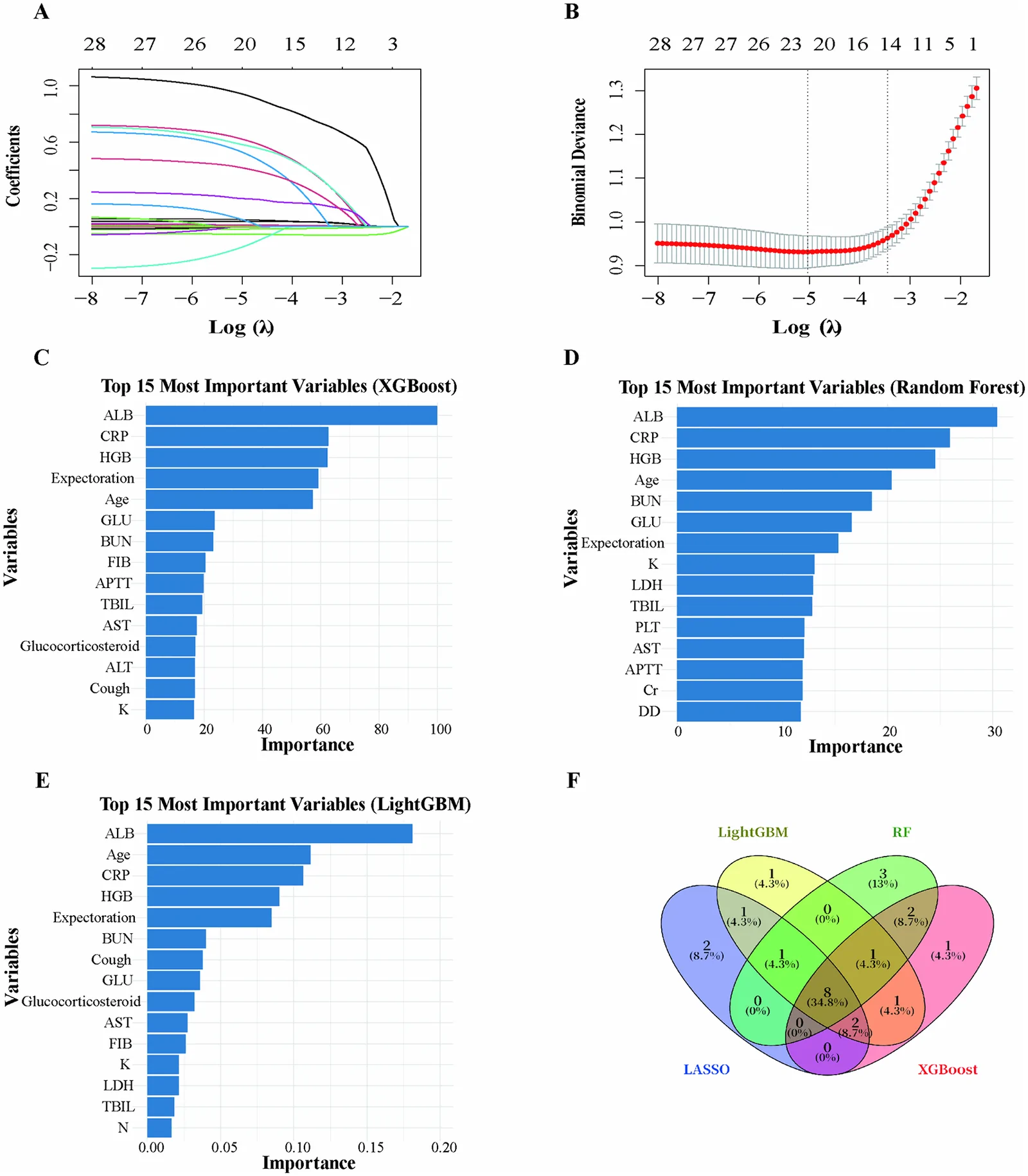
Selecting variables through four machine learning algorithms. (A) LASSO coefficient profiles of the 28 variables of the clinical diagnosis prediction model in SFTS patients. (B) Cross-validation results of the clinical diagnosis prediction model for diagnosis of superinfection in SFTS patients. (C) Top 15 variables in XGBoost. (D) Top 15 variables in RF. (E) Top 15 variables in LightGBM. (F) Intersection of variables selected by four algorithms-Venn diagram. Abbreviations: SFTS, severe fever with thrombocytopenia syndrome; LASSO, least absolute shrinkage and selection operator regression; XGBoost, extreme gradient boosting; RF, random forest; LightGBM, light gradient boosting machine.

To establish a model for predicting superinfection in a clinical setting, we applied LR, LASSO regression, RF, LightGBM, and XGBoost to identify the most effective predictors. In the training cohort, we first conducted univariate logistic regression and selected 28 indicators related to the outcome from 36 indicators (*p* < 0.05, S1 Table). Subsequently, we apply LASSO regression to refine the selection of predictors. Fourteen of the twenty-eight predictors (age, use of glucocorticoids, ALB, AST, LDH, BUN, GLU, CRP, HGB, DM, cough, confusion, expectoration, bleeding) were identified as potential predictors by LASSO regression analysis (Fig 2A and 2B). Given that different algorithms introduce inherent randomness, the number of trees in RF was set to 500, for XGBoost, the learning rate was adjusted to 0.1, and the maximum tree depth to 3 for optimal classification performance. In LightGBM, the learning rate and maximum tree depth were set to 0.01 and 3, respectively. The feature importance scores derived from these parameter settings were used as rankings for the respective methods (Fig 2C–2E).

As shown in Fig 2C–2E, the top 15 most important variables were screened out by RF, LightGBM, and XGBoost machine algorithms, respectively. Finally, we selected the top 15 variables from RF, LightGBM, and XGBoost based on their importance ranking and identified their intersection with the 14 variables selected in the previous LASSO regression. Ultimately, 8 key indicators were identified as predictors for constructing the superinfection model: age, ALB, AST, BUN, GLU, CRP, HGB, and expectoration (Fig 2F).

### Prediction model construction and validation

**Fig 3:**
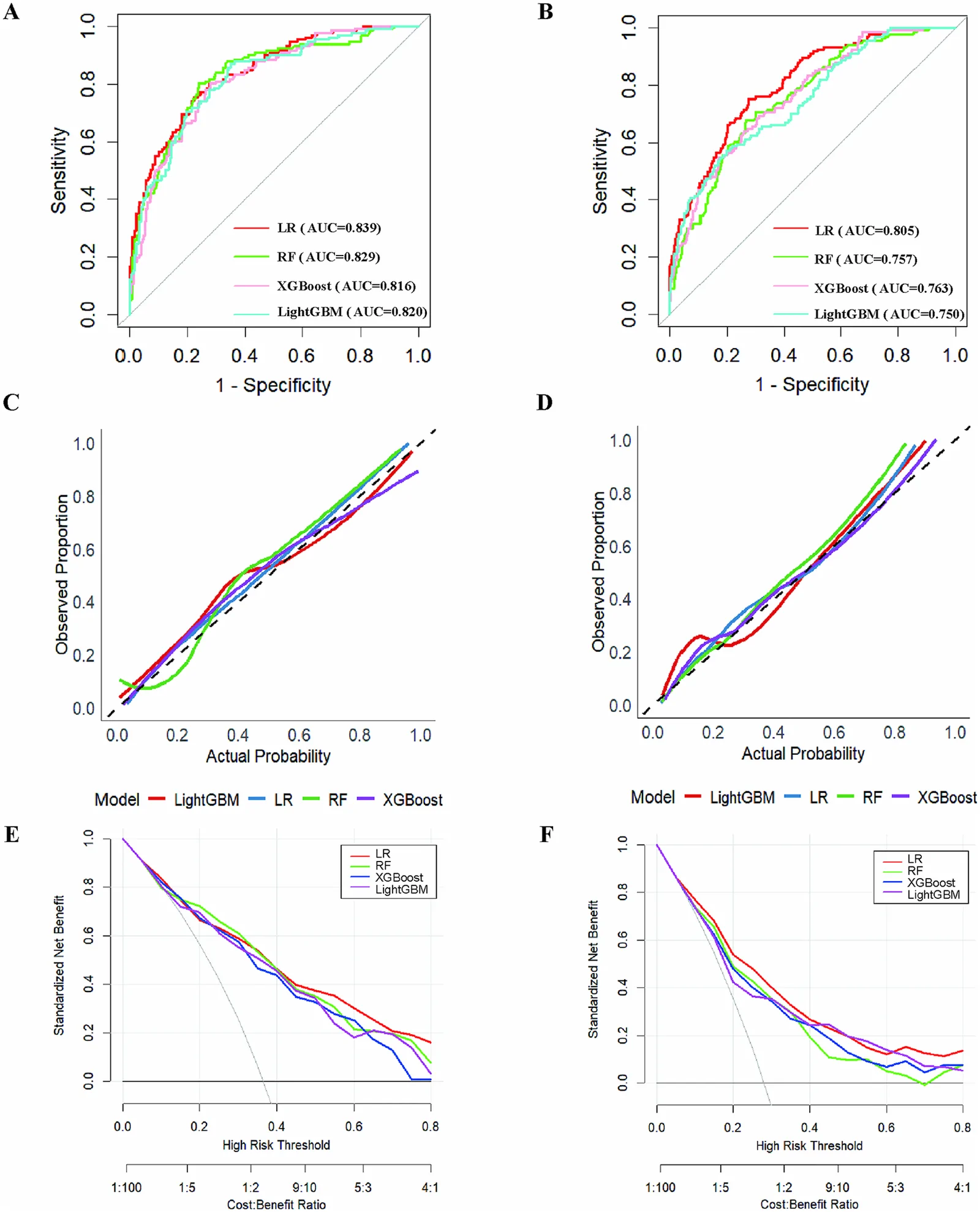
Receiver operating characteristic curve, calibration curve and decision curve for 4 machine learning algorithms. (A) Receiver operating characteristic curve for the internal validation cohort. (B) Receiver operating characteristic curve for the external validation cohort. (C) Calibration curve for the internal validation cohort. (D) Calibration curve for the external validation cohort. (E) Decision curve for the internal validation cohort. (F) Decision curve for the external validation cohort. Abbreviations: SFTS, severe fever with thrombocytopenia syndrome; ROC, receiver operating characteristic curve; DCA, decision curve analysis.

Based on the 8 predictors, LR, RF, LightGBM, and XGBoost were employed to construct and validate predictive models, respectively. As illustrated in Fig 3A and 3B, LR achieved the highest performance both on the internal and external validation sets, with AUC values of 0.839 (95% CI: 0.797–0.880) versus 0.805 (95% CI: 0.764–0.847). RF had an internal validation set AUC of 0.829, which dropped to 0.757 on external validation. XGBoost also showed good internal validation performance, but experienced a notable decline in external validation, dropping from 0.816 to 0.763. LightGBM achieved a performance of 0.820 on the internal validation set, but its performance dropped significantly to 0.750 on the external validation set. With a minimal difference of only 0.034 between the internal and external validation set performance, LR exhibited strong generalization ability and proved to be the most stable and highest-performing method in the crucial validation stage. In terms of clinical utility, LR yielded a positive net benefit across a broad range of clinically relevant thresholds (approximately 0.1 to 0.8) (Fig 3E and 3F). Additionally, LR demonstrated excellent specificity in both internal (0.904) and external (0.930) validation, indicating a low rate of false-positive predictions. The Brier score of LR remained stable at 0.154 in internal validation and 0.151 in external validation, suggesting good calibration and minimal overfitting. Taken together, these findings support LR as the final predictive model. It offered superior discrimination, clinical practicality, consistent generalisability, and favourable performance across validation sets (S2 Table).

**Fig 4:**
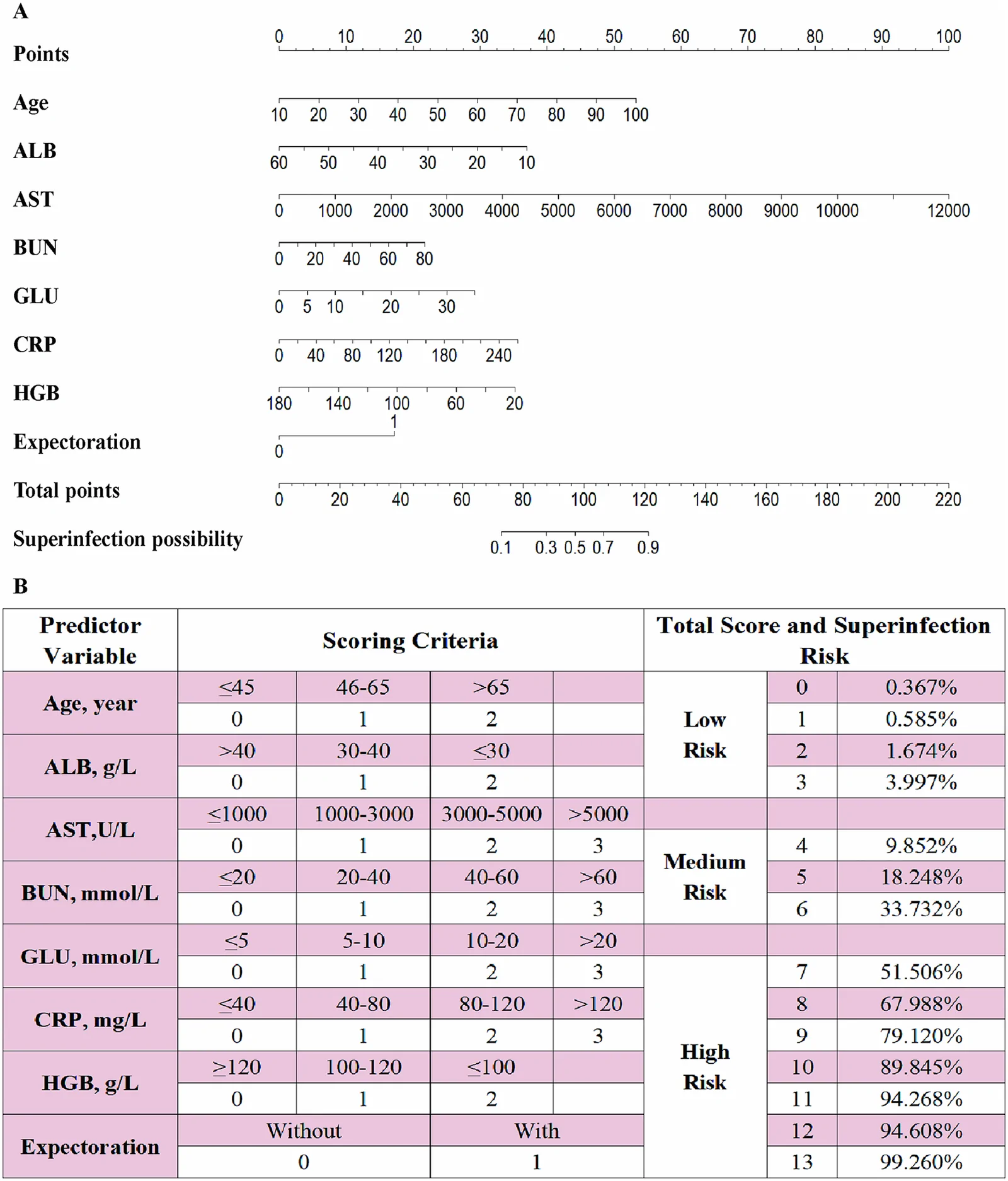
The nomogram and scoring scale for predicting superinfection in SFTS patients. (A) The nomogram for predicting superinfection in SFTS patients. (B) The scoring scale for predicting superinfection in SFTS patients. Abbreviations: SFTS, severe fever with thrombocytopenia syndrome; ALB, albumin; AST, aspartate aminotransferase; GLU, glucose; CRP, c-reactive protein; HGB, haemoglobin; BUN, urea nitrogen.

Due to its superior performance, the LR model was selected for final deployment. This model, utilizing eight predictors (Age, ALB, AST, BUN, GLU, CRP, HGB, and expectoration), was subsequently visualized as a nomogram (Fig 4A). To enhance clinical application, the model’s predictors were subsequently segmented into hierarchical variables and rescored according to the nomogram. A novel scale scoring from 0 to 13 points was finally developed. The probability of superinfection for each score was determined through LR analysis to quantitatively stratify the superinfection risk in SFTS patients (Fig 4B). According to the scale, the risk of superinfection was categorized into three levels: low risk (≤3), medium risk (4–6), and high risk (7–13)(Fig 4B). These cutpoints were derived using a data-driven quantile-based method based on the score distribution, ensuring reproducible and non-arbitrary risk classification. The predictive performance of the simplified scoring system was subsequently compared with the nomogram, demonstrating comparable discrimination with only a slight reduction in AUC (S3 Table).

### Prospective validation of the model in a real-world clinical setting

**Fig 5:**
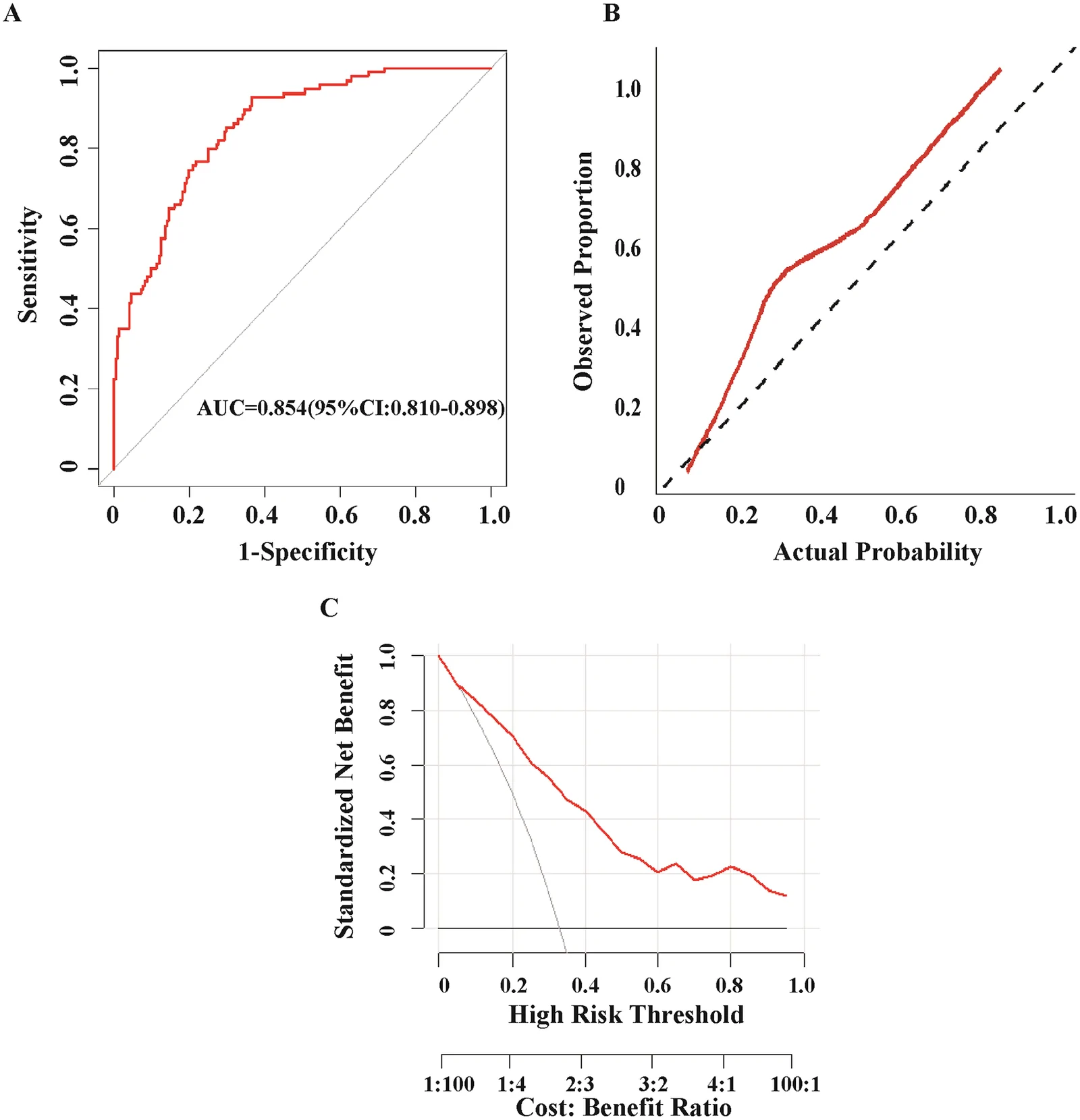
Performance of superinfection prediction model in a real-world prospective cohort. (A) ROC for LR model in the real-world cohort. (B) Calibration curve for LR model in the real-world cohort. (C) DCA for LR model in the real-world cohort. Abbreviations: ROC, receiver operating characteristic curve; LR, logistic regression; DCA, decision curve analysis.

The model was then prospectively validated in real-world clinical settings at two hospitals throughout 2025, where it was used to dynamically assess individual patient risk scores. The model showed strong performance in the prospective, real-world setting. As shown in Fig 5A, the model achieved excellent discrimination, with an AUC of 0.854 (95% CI: 0.810–0.898). It also exhibited strong reliability, with good calibration displayed in Fig 5B. The DCA in Fig 5C showed a greater benefit compared to either ‘no treatment’ or ‘all treatment’ in the range of 20% -100%. In brief, the model demonstrated high stability and superior predictive performance in a real-world clinical setting.

### Comparison of nomogram and simplified scoring scale

As shown in S3 Table, both the nomogram and the scoring scale exhibited excellent fitting goodness and predictive performance across all datasets. Under the optimal cut-off values, the nomogram achieved a strong negative predictive value (NPV) of 78.6% in the prospective validation, indicating a robust capability for ruling out low-risk patients. While the nomogram showed a slightly higher AUROC in the training set (0.839 vs. 0.809), reaching 0.843 in the prospective validation. This confirms that simplifying the model into a scoring system does not substantially reduce predictive performance. Notably, in the prospective cohort, the scoring scale demonstrated a remarkably higher specificity (95.3% vs. 88.5%) and a significantly stronger positive likelihood ratio (9.482 vs. 4.433), indicating a superior ability to rule in high-risk patients while minimizing false positives. By simplifying complex coefficients into an intuitive integer-based system (Cut-off: 6.5), the scoring scale offers a more accessible and rapid decision-making tool for clinicians without compromising diagnostic accuracy or predictive stability.

## Discussion

SFTS is an emerging tick-borne hemorrhagic fever with high mortality rates that has garnered increasing attention worldwide. Superinfections can prolong the hospitalization time and increase the mortality of SFTS patients [23,24]. In this study, we revealed that the incidence of superinfection in SFTS patients was 33.57%. We observed a notably high proportion of fungal superinfections, with *Aspergillus* species being particularly prevalent. Immunosuppression caused by SFTSV infection, glucocorticoid use, and ICU exposure has all been reported as risk factors for invasive aspergillosis [25–27]. Besides, we report 4 cases of *M. tuberculosis* infection in SFTS patients. However, due to the lack of baseline pre-infection screening and the rapid progression of SFTS, we were unable to definitively differentiate between newly acquired active TB and the reactivation of latent tuberculosis (LTBI). When diagnosing superinfection in SFTS patients is uncertain, adherence to antibiotic stewardship programs often declines as clinicians prioritize life-saving interventions.

Early identification of SFTS patients with superinfection can help clinicians focus on timely management and prevent the overuse of antibiotics. In this study, we employed five machine learning algorithms with a cross-selection approach to identify the most robust and non-redundant predictors. This strategy effectively reduced the initial 36 candidate variables to 8 final predictive factors for superinfection. The eight predictors capture complementary dimensions of superinfection risk. CRP reflects systemic inflammatory activity. Age, ALB, HGB, and GLU summarise the patient’s overall physiological condition. AST and BUN reflect hepatic and renal dysfunction. Expectoration represents the dominant clinical manifestation. Together, these variables enable a multi-dimensional, bedside assessment of superinfection risk. These predictors are easily accessible factors, independent of viral parameters, making them applicable even in primary healthcare settings.

While previous studies have investigated superinfection in SFTS, most were limited by small-sample size and single-center retrospective designs [9,28]. Furthermore, these studies often prioritized internal validation, lacking assessment in external cohorts [29]. To address these gaps, our study gathered 12 years of consecutive clinical data from four hospitals. We employed four machine learning algorithms to minimise overfitting and enhance generalisability. The resulting prediction model for superinfection in SFTS patients showed high accuracy in both the external and prospective real-world validation cohorts. Building on the predictive results visualised in the nomogram, we further developed a novel 3-tier risk scoring scale. The scale enables simple, intuitive bedside stratification of superinfection risk in SFTS patients. To our knowledge, this is one of the first studies to establish a superinfection risk stratification system for SFTS patients.

However, there were several limitations. A primary limitation of this study is that all datasets and external validation sets were obtained from hospitals in Anhui Province. No independent external validation from regions outside Anhui was conducted, limiting geographic generalizability. Future research should aim to validate the model in multicenter cohorts across different geographic regions. Second, our study did not develop specific diagnostic models for bacterial or fungal superinfections, owing to the presence of dual-pathogen infections in some patients, which complicated differential diagnosis. Additionally, our primary goal was to distinguish superinfection from non-superinfection at the earliest stage to help control antimicrobial overuse in SFTS patients. Third, we did not analyze the bacterial resistance profile because some pathogens identified via NGS cannot provide a resistance phenotype.

In conclusion, this evidence-based, factor-weighted, accurate scoring scale provides clinicians with an effective tool to quickly stratify superinfection risk in SFTS patients beyond pathogen.

## Supporting information

S1 Fig Microbiological aetiologies of SFTS patients with superinfection. (A) Distribution of Gram-negative bacteria. (B) Distribution of Gram-positive bacteria. (C) Distribution of fungi. Abbreviations: *K. pneumoniae*, *Klebsiella pneumoniae*; *E. coli*, *Escherichia coli*; *P. aeruginosa*, *Pseudomonas aeruginosa*; *S. maltophilia, Stenotrophomonas maltophilia*; *E. cloacae*, *Enterobacter cloacae*; *H. influenzae*, *Haemophilus influenzae*; *B. cereus, Bacillus cereus*; *M. luteus*, *Micrococcus luteus*; *A. fumigatus*, *Aspergillus fumigatus*; *A. flavus*, *Aspergillus flavus*.
(TIF)

S1 Table Univariate logistic regression results of the training set. Abbreviations: ALB, albumin; GLO, globulin; TBIL, total bilirubin; ALT, alanine aminotransferase; AST, aspartate aminotransferase; LDH, lactate dehydrogenase; BUN, blood urea nitrogen; Cr, creatinine; K, potassium; Na, sodium; GLU, glucose; CRP, C-reactive protein; WBC, white blood cell; N, neutrophil count; HGB, hemoglobin; PLT, platelet count; APTT, activated partial thromboplastin time; FIB, fibrinogen; DD, d-dimer; CK, creatinine kinase; CK-MB, creatine kinase MB; DM, diabetes mellitus; COPD, chronic obstructive pulmonary disease; CI, confidence interval.
(DOCX)

S2 Table Comparative performance of machine learning models in validation sets. (DOCX)

S3 Table Fitting goodness and predictive performance of the nomogram and scoring scale. (DOCX)

S4 Table Hyperparameter search spaces for each model. (DOCX)
