# Supplementary material for: A clinical scoring system for the early identification of superinfections in patients with severe fever with thrombocytopenia syndrome: An ambispective multicentre cohort study
Source: PLoS Negl Trop Dis. 2026 Sep 10;20(9):e0014696. doi: 10.1371/journal.pntd.0014696 (PMC13561336; doi:10.1371/journal.pntd.0014696)
Supplement: Supplementary file 1 [file pntd.0014696.s002.docx]

**S1 Table. Univariate logistic regression results of the training set.**

| **Variable** | **OR** | **95% CI** | ***P*** |
| --- | --- | --- | --- |
| Sex | 0.850 | 0.640-1.130 | 0.269 |
| Age | 1.060 | 1.040-1.070 | <0.001 |
| Glucocorticosteroid use | 2.410 | 1.770-3.270 | <0.001 |
| Ststolicpressure | 0.956 | 0.829-1.103 | 0.540 |
| ALB | 0.830 | 0.800-0.860 | <0.001 |
| GLO | 1.030 | 1.000-1.060 | 0.029 |
| TBIL | 1.381 | 1.183-1.612 | <0.001 |
| ALT | 1.233 | 1.050-1.449 | 0.011 |
| AST | 1.940 | 1.514-2.487 | <0.001 |
| LDH | 1.685 | 1.391-2.041 | <0.001 |
| BUN | 1.100 | 1.070-1.140 | <0.001 |
| Cr | 1.524 | 1.277-1.818 | <0.001 |
| K | 0.640 | 0.480-0.840 | 0.001 |
| Na | 0.960 | 0.930-0.990 | 0.004 |
| GLU | 1.140 | 1.100-1.190 | <0.001 |
| CRP | 2.036 | 1.685-2.459 | <0.001 |
| WBC | 1.000 | 0.930-1.070 | 0.972 |
| N | 0.990 | 0.910-1.080 | 0.891 |
| HGB | 0.475 | 0.402-0.561 | <0.001 |
| PLT | 0.563 | 0.462-0.684 | <0.001 |
| APTT | 1.503 | 1.292-1.748 | <0.001 |
| FIB | 1.160 | 1.050-1.290 | 0.005 |
| DD | 1.060 | 1.030-1.080 | <0.001 |
| CK | 1.163 | 1.007-1.343 | 0.040 |
| CKMB | 1.717 | 1.165-2.530 | 0.006 |
| DM | 2.550 | 1.600-4.070 | <0.001 |
| COPD | 2.180 | 0.960-4.920 | 0.061 |
| Fever | 2.120 | 0.690-6.440 | 0.187 |
| Gastrointestinal symptoms | 1.020 | 0.760-1.370 | 0.877 |
| Lymphadenopathy | 1.230 | 0.910-1.680 | 0.183 |
| Cough | 3.920 | 2.830-5.420 | <0.001 |
| Confusion | 3.260 | 2.340-4.550 | <0.001 |
| Expectoration | 5.000 | 3.480-7.170 | <0.001 |
| Bleeding | 3.120 | 2.220-4.390 | <0.001 |
| Limbtremor | 2.110 | 1.390-3.190 | <0.001 |
| Coma | 2.590 | 1.600-4.200 | <0.001 |

Abbreviations: ALB, albumin; GLO, globulin; TBIL, total bilirubin; ALT, alanine aminotransferase; AST, aspartate aminotransferase; LDH, lactate dehydrogenase; BUN, blood urea nitrogen; Cr, creatinine; K, potassium; Na, sodium; GLU, glucose; CRP, C-reactive protein; WBC, white blood cell; N, neutrophil count; HGB, hemoglobin; PLT, platelet count; APTT, activated partial thromboplastin time; FIB, fibrinogen; DD, d-dimer; CK, creatinine kinase; CK-MB, creatine kinase MB; DM, diabetes mellitus; COPD, chronic obstructive pulmonary disease; CI, confidence interval.
