# Supplementary material for: A clinical scoring system for the early identification of superinfections in patients with severe fever with thrombocytopenia syndrome: An ambispective multicentre cohort study
Source: PLoS Negl Trop Dis. 2026 Sep 10;20(9):e0014696. doi: 10.1371/journal.pntd.0014696 (PMC13561336; doi:10.1371/journal.pntd.0014696)
Supplement: Supplementary file 2 [file pntd.0014696.s003.docx]

S2 Table. Comparative performance of machine learning models in validation sets

| **Evaluation indicators** | **Internal validation** | | | |  | **External validation** | | | |  | **Prospective validation** |
| --- | --- | --- | --- | --- | --- | --- | --- | --- | --- | --- | --- |
|  | **LR** | **RF** | **XGBoost** | **LightGBM** |  | **LR** | **RF** | **XGBoost** | **LightGBM** |  | **LR** |
| AUC (95%CI) | 0.839 (0.797-0.880) | 0.829 (0.784-0.873) | 0.816 (0.772-0.860) | 0.820 (0.775-0.864) |  | 0.805 (0.764-0.847) | 0.757 (0.710-0.803) | 0.763 (0.717-0.809) | 0.750 (0.701-0.798) |  | 0.854 (0.810-0.898) |
| Accuracy | 0.774 | 0.760 | 0.754 | 0.760 |  | 0.773 | 0.754 | 0.754 | 0.781 |  | 0.758 |
| Sensitivity | 0.550 | 0.557 | 0.565 | 0.595 |  | 0.368 | 0.226 | 0.316 | 0.353 |  | 0.351 |
| Specificity | 0.904 | 0.877 | 0.864 | 0.855 |  | 0.930 | 0.959 | 0.924 | 0.947 |  | 0.958 |
| Positive Predictive Value, PPV | 0.766 | 0.723 | 0.705 | 0.703 |  | 0.671 | 0.681 | 0.618 | 0.723 |  | 0.805 |
| Negative Predictive Value, NPV | 0.777 | 0.775 | 0.776 | 0.786 |  | 0.791 | 0.761 | 0.776 | 0.790 |  | 0.750 |
| Youden's index | 0.532 | 0.560 | 0.521 | 0.519 |  | 0.477 | 0.411 | 0.383 | 0.373 |  | 0.559 |
| Brier | 0.154 | 0.160 | 0.167 | 0.165 |  | 0.151 | 0.167 | 0.164 | 0.164 |  | 0.154 |
