# Supplementary material for: A clinical scoring system for the early identification of superinfections in patients with severe fever with thrombocytopenia syndrome: An ambispective multicentre cohort study
Source: PLoS Negl Trop Dis. 2026 Sep 10;20(9):e0014696. doi: 10.1371/journal.pntd.0014696 (PMC13561336; doi:10.1371/journal.pntd.0014696)
Supplement: Supplementary file 3 [file pntd.0014696.s004.docx]

S3 Table. Fitting goodness and predictive performance of the nomogram and scoring scale.

|  | **Training** | |  | **Internal validation** | |  | **External validation** | |  | **Prospective validation** | |
| --- | --- | --- | --- | --- | --- | --- | --- | --- | --- | --- | --- |
| **Evaluation indicators** | **Nomogram** | **Scoring scale** |  | **Nomogram** | **Scoring scale** |  | **Nomogram** | **Scoring scale** |  | **Nomogram** | **Scoring scale** |
| Nagelkerke R^2^ | 0.441 | 0.381 |  | 0.434 | 0.339 |  | 0.339 | 0.264 |  | 0.404 | 0.381 |
| Cut-off Value | 0.35 | 6.5 |  | 0.35 | 6.5 |  | 0.35 | 6.5 |  | 0.35 | 6.5 |
| AUROC (95% CI) | 0.839 (0.811-0.866) | 0.809 (0.779-0.839) |  | 0.839 (0.797-0.880) | 0.799 (0.753-0.846) |  | 0.805 (0.764-0.847) | 0.766 (0.722-0.810) |  | 0.854 (0.810-0.898) | 0.843 (0.797-0.888) |
| Youden’s index | 0.514 | 0.453 |  | 0.524 | 0.456 |  | 0.395 | 0.268 |  | 0.396 | 0.400 |
| Sensitivity (%) | 74.5 | 63.3 |  | 74.8 | 61.8 |  | 55.6 | 37.6 |  | 51.1 | 44.7 |
| Specificity (%) | 76.9 | 82.0 |  | 77.6 | 83.8 |  | 83.9 | 89.2 |  | 88.5 | 95.3 |
| Crude agreement (%) | 76.1 | 75.3 |  | 76.6 | 75.8 |  | 76.0 | 74.7 |  | 76.1 | 78.6 |
| Adjusted agreement (%) | 49.7 | 45.8 |  | 51.0 | 46.6 |  | 39.9 | 29.9 |  | 42.3 | 45.2 |
| Positive predictive value (%) | 64.2 | 66.2 |  | 65.8 | 68.6 |  | 57.4 | 57.5 |  | 68.6 | 82.4 |
| Negative predictive value (%) | 84.4 | 80.1 |  | 84.3 | 79.3 |  | 82.9 | 78.6 |  | 78.6 | 77.8 |
| Positive likelihood ratio | 3.230 | 3.523 |  | 3.344 | 3.810 |  | 3.460 | 3.475 |  | 4.433 | 9.482 |
| Negative likelihood ratio | 0.332 | 0.448 |  | 0.324 | 0.456 |  | 0.529 | 0.700 |  | 0.553 | 0.581 |
