# Supplementary material for: A clinical scoring system for the early identification of superinfections in patients with severe fever with thrombocytopenia syndrome: An ambispective multicentre cohort study
Source: PLoS Negl Trop Dis. 2026 Sep 10;20(9):e0014696. doi: 10.1371/journal.pntd.0014696 (PMC13561336; doi:10.1371/journal.pntd.0014696)
Supplement: Supplementary file 4 [file pntd.0014696.s005.docx]

**S4 Table. Hyperparameter search spaces for each model.**

| Model | Hyperparameters | Search Space (Consistent with Code) |
| --- | --- | --- |
| Logistic Regression (LR) | No hyperparameter tuning (baseline model) | Trained with glm(family = "binomial"), classification threshold = 0.5. Used as a linear baseline without additional |
| Random Forest (RF) | Number of trees (ntree); Number of features per split (mtry) | mtry: 1–√(n_features);  ntree: 50–1000 (step = 50), optimal selected by minimizing OOB error |
| LightGBM | num_leaves; max_depth; learning_rate; n_estimators; min_data_in_leaf; lambda_l1; lambda_l2 | num_leaves: c(15,31); max_depth: c(-1,1,3); learning_rate: c(0.1,0.2); n_estimators: c(50); min_data_in_leaf: c(30); lambda_l1/lambda_l2: c(0,1) |
| XGBoost | max_depth; eta; nrounds | max_depth: c(2, 3, 4, 5); eta: c(0.01, 0.1, 0.2); nrounds:c(50, 100, 150); |
